# ELISPOT Refinement Using Spot Morphology for Assessing Host Responses to Tuberculosis

**DOI:** 10.3390/cells1010005

**Published:** 2012-03-13

**Authors:** Laura S. Sibley, Andrew D. White, Alice Marriott, Michael J. Dennis, Ann Williams, Philip D. Marsh, Sally A. Sharpe

**Affiliations:** Microbiological Services, Health Protection Agency Porton, Porton Down, Salisbury, Wiltshire SP4 0JG, UK; E-Mails: Laura.Sibley@hpa.org.uk (L.S.S.); Andrew.White@hpa.org.uk (A.D.W.); alice.marriott@hpa.org.uk (A.M.); mike.dennis@hpa.org.uk (M.J.D); ann.rawkins@hpa.org.uk (A.W.); phil.marsh@hpa.org.uk (P.D.M.)

**Keywords:** ELISPOT, IFNγ, tuberculosis, BCG

## Abstract

Tuberculosis is a global health problem. The *Mycobacterium bovis* Bacille Calmette Guerin (BCG) vaccine has variable efficacy (0-80%) so there is a drive to develop novel vaccines. The cytokine, interferon gamma (IFNγ), is an essential component of the protective response to *M. tuberculosis* (*M. tb*) infection and is also produced in response to BCG vaccination. Induction of an IFNγ response is used as a biomarker of successful vaccination in the assessment of new tuberculosis (TB) vaccines. The IFNγ ELISPOT assay provides an important tool for TB research. It is used for both the diagnosis of infection (T.Spot assay), and for the evaluation of the immunogenicity of new TB vaccine candidates in human clinical trials, in the non-human primate (NHP) model of TB infection studies. The ELISPOT assay captures IFNγ produced by peripheral blood mononuclear cells (PBMCs) following specific stimulation, onto a membrane so individual cells can be enumerated and the frequency of responding cells determined. Hence spot forming units (SFU) per 10^6^ cells provide the traditional measure for ELISPOT assays. The discriminatory power of SFU is limited. In some situations, the number of SFU in BCG vaccinated, and unvaccinated, subjects was found to be similar, although the spots were observed to be larger in vaccinated subjects. Spot size potentially provides a measure of the quantity of cytokine produced by individual cells. The AID ELISPOT plate reader software used to determine frequency of spots also has the capability to determine the size of each spot. Consideration of spot size in combination with spot forming units was investigated in our studies of BCG immunogenicity. This additional readout was found to enhance the discriminatory power of the ELISPOT assay, and provide more information on the immune response to BCG vaccination and infection with *M.tb*.

## 1. Introduction

Tuberculosis (TB) in humans is caused by infection with *Mycobacterium tuberculosis* (*M tb*) and is one of the leading global causes of death from a single infectious agent, with an estimated 8.8 million new cases worldwide and 1.4 million deaths in 2011 [1]. A third of the world’s population is estimated to be latently infected with *M. tb*, and these people carry a 10% lifetime risk of developing active life-threatening disease [2] which is increased to 10% per year in the event of co-infection with human immunodeficiency virus [3,4]. The global TB pandemic has been further exacerbated by the emergence of drug resistant strains of *M. tuberculosis* which render treatment less effective. There is an urgent need for novel vaccines and other therapeutics to reduce the number of cases.The only licensed TB vaccine, *Mycobacterium bovis* Bacille Calmette Guerin (BCG), is administered to neonates in high-risk populations as part of the WHO Expanded Programme on Immunisation. BCG consistently protects against TB meningitis and disseminated TB in childhood [5] but its efficacy wanes with time and it affords only variable protection against pulmonary disease. A new, more effective TB vaccine is a major global health priority and is an important part of the WHO STOP TB partnership strategy. 

The T-cell response is important in control of TB infection, as illustrated by the large incidence of TB infection in patients who are HIV^+^, and lack sufficient numbers of CD4^+^ cells to be able to tackle the infection effectively [5]. The Th1 cytokine IFNγ has been shown to be a vital component of the protective immune response to TB, and there is evidence that individuals lacking the IFNγ gene are far more susceptible to TB infection [6]. IFNγ is involved in several mechanisms of protective immunity including activation of macrophages and NK cells and in T-cell differentiation and has therefore been suggested as a correlate of protection. 

However, it has been shown [7,8] that IFNγ alone does not correlate with protection, but IFNγ remains an important component of the overall immune response required to combat infection with tuberculosis. IFNγ release assays (IGRAs) are therefore very important tools for measuring the immune response to *M. tb* infection (including diagnosis of infection) and for monitoring the impact of vaccination. The IFNγ ELISPOT assay is widely used and measures the frequency of cells which produce IFNγ in response to stimulation by specific antigens. A variant of the ELISPOT assay, the T-spot test uses TB antigens ESAT-6 and CFP10 to diagnose infection with TB [9]. The ELISPOT assay is widely used in research to monitor the Th1 response in clinical trials of new TB vaccines [10] and pre-clinical vaccine evaluation studies [7]. The ELISPOT assay shows increased sensitivity over other IGRAs as it is able to detect IFNγ release by single cells. 

The traditional ELISPOT assay readout is expressed as spot forming units (SFU) per 10^6^ cells. SFU is a well established measurement, but does not correlate with protection [7] and it can lack discriminatory power. We have observed that the sizes of the spots generated in the ELISPOT assay may vary, and that after vaccination or infection, the size of the spots increases noticeably. The size of the spot may either relate to the quantity of cytokine being excreted by individual cells, or the actual morphology of the cells, and this could potentially provide additional information on the immune response which is not taken in to account when SFU alone are counted.

In this study the effect of incorporating a measure of spot size into the ELISPOT assay readout in combination with quantity of spots was investigated for potential to (a) enhance the discriminatory power of the ELISPOT and (b) provide more information on the immune response to BCG vaccination or *M. tb* infection. 

## 2. Results and Discussion

### 2.1. Investigation of the Utility of an ELISPOT Readout Incorporating Spot Size for Evaluation of Immune Responses Induced by BCG Vaccination

**Figure 1:**
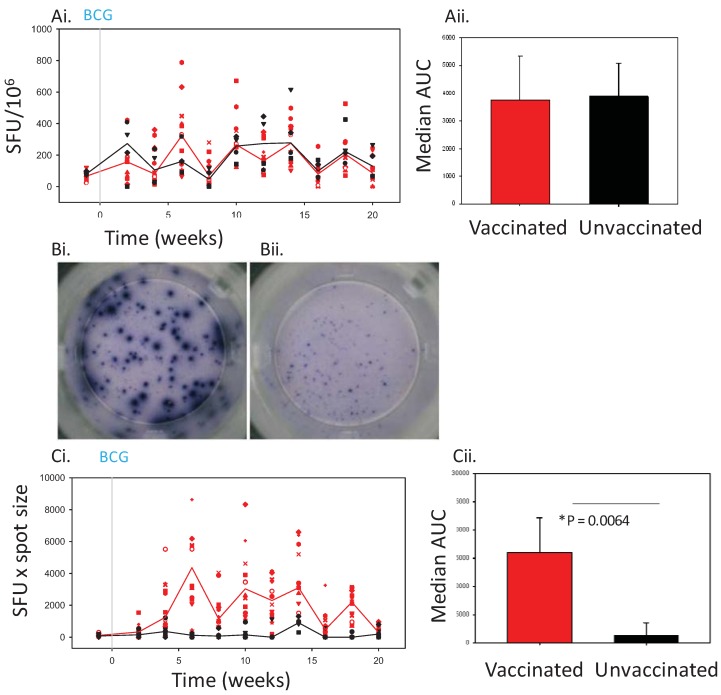
The IFNγ response to *Mycobacterium bovis* Bacille Calmette Guerin (BCG) vaccination in Mauritian cynomolgus macaques measured by ELISPOT.Panel Ashows the frequency of purified protein derivative (PPD)-specific IFN-γ secreting cells in peripheral blood mononuclear cells (PBMC) measured as spot forming units (SFU)/10^6^ in individual BCG vaccinated n = 16 (red symbols) and unvaccinated n = 4 (black symbols) macaques and the median response in each group (BCG vaccinated group, red line, unvaccinated group, black line) (**Ai**) and the median area under the SFU curve (area under the curve (AUC)) calculated for the BCG vaccinated (red bar) and unvaccinated (black bar) study groups and compared using a Mann Whitney test (**Aii**). Panel B shows images of spots which developed in wells containing PBMC stimulated with PPD from a BCG vaccinated macaque (**Bi**) and an unvaccinated macaque (**Bii**). Panel C shows the IFNγ response profiles determined using spot size x quantity from individual BCG vaccinated (red symbols) and unvaccinated (black symbols) macaques and the median response in each group (BCG vaccinated group, red line, unvaccinated group, black line) (**Ci**), and the median area under the spot size x quantity curves (AUC) calculated for BCG vaccinated (red bar) and unvaccinated (black bar) study groups and compared using a Mann Whitney test (**Cii**).

Data from two non-human primate (NHP) studies were used to investigate spot size as an additional measurement. The frequency of peripheral blood mononuclear cells (PBMC) capable of producing IFNγ in response to stimulation with purified protein derivative (PPD), in blood collected from Mauritian cynomolgus macaques in Study 1, over the first 20 weeks after vaccination with BCG, enumerated as SFU was found to be similar to the frequency determined in PBMC from animals that were not vaccinated (Figure 1Ai). Therefore, the immune profiles of vaccinated and unvaccinated animals could not be distinguished based on the frequency of spots alone. Comparison of the area under the curves derived from the response profiles based on SFU in BCG vaccinated and unvaccinated animals using a Mann Whitney test (Figure 1A) confirmed the lack of difference between the groups (p = 0.8557) (Figure 1Ai). 

The morphology of spots observed in wells set up with PBMC isolated from BCG vaccinated animals was different to that seen in wells set up with PBMC from unvaccinated animals. Fig1B shows images of two wells which contain the same number of spots, however the spots produced in well (Bi) by PBMC from a BCG vaccinated animal are markedly larger than those produced in well (Bii) by PBMC from an unvaccinated animal. The larger spot sizes in well (Bi) suggest PBMC from the BCG vaccinated macaque produced more IFNγ than PBMC from the unvaccinated macaque when stimulated with PPD. 

When a measurement of spot count x average spot size was used to compare the IFNγ response in vaccinated and unvaccinated macaques, a response of increased magnitude with a peak at week 6 was revealed in the animals that received the BCG vaccine. AUC and Mann-Whitney analysis confirmed the response profiles to be significantly different (p = 0.0064 (Figure 1C)).

### 2.2. Verification of the Improved Discriminatory Power Provided by Incorporation of Spot Size

**Figure 2:**
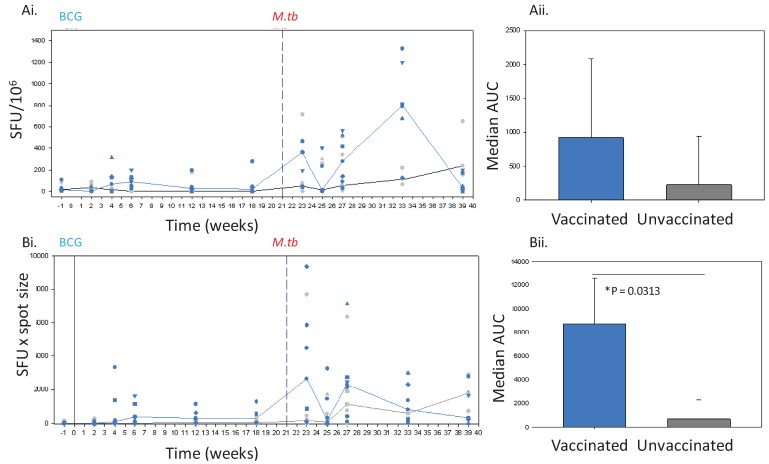
The IFNγ response to BCG vaccination (solid line) and challenge with *M. tb* (dashed line) in Chinese cynomolgus macaques measured by ELISPOT. Panel Ashows the frequency of PPD-specific IFN-γ secreting cells in PBMC measured as SFU/10^6^ in individual BCG vaccinated n = 6 (blue symbols) and unvaccinated n = 6 (grey symbols) macaques and the median response in each group (BCG vaccinated group, blue line, unvaccinated group, black line) (**Ai**) and the median area under the SFU curves (AUC) calculated for BCG vaccinated (blue bar) and unvaccinated (grey bar) study groups during the vaccination phase and compared using a Mann Whitney test (**Aii**). Panel B shows the IFNγ response profiles determined using spot size x quantity from individual BCG vaccinated (blue symbols) and unvaccinated (grey symbols) macaques and the median response in each group (BCG vaccinated group, blue line, unvaccinated group, grey line) (**Bi**), and the median area under the spot size x quantity curves (AUC) calculated for BCG vaccinated (blue bars) and unvaccinated (grey bars) study groups during the vaccination phase and compared using a Mann Whitney test (**Bii**).

The response made by the PPD-specific IFNγ secreting cell population induced in the peripheral blood of Chinese cynomolgus macaques following vaccination with BCG was investigated using both ELISPOT readouts in Study 2. The frequency of PPD-specific, IFNγ producing PBMC, enumerated using SFU, was greater in BCG vaccinated macaques than in macaques that were not vaccinated during the 18 weeks of study (Figure 2A). However, comparison of the area under the curves derived from the response profiles based on the SFU measurement using a Mann Whitney test found that the difference in the response profiles did not reach statistical significance (p = 0.1735). In contrast, the response profile measured by spot count x area (Figure 2B) revealed an increase in IFNγ response after BCG vaccination that was significantly different (p = 0.0131) to the response in the unvaccinated animals. Therefore, both measures were able to demonstrate that BCG vaccination stimulated an increase in IFNγ production but the use of the spot count x area measurement had improved discriminatory power. A peak IFNγ response 6 weeks after vaccination was identified using both response measures and although the frequency of SFU decreased after week 6, the profile derived from spot count x area suggested that the response was maintained for 18 weeks. 

### 2.3. Analysis of the IFNy Response Induced in Chinese Cynomolgus Macaques Following Infection with M.tb Using the Spot Size x Quantity and SFU ELISPOT Readouts

PPD-specific IFNγ response profiles determined using frequency of spots alone (Figure 2A) and spot count x size (Figure 2B) both showed that the responses made in the period following aerosol exposure to *M. tb*, were greater than those detected prior to challenge in both BCG vaccinated and unvaccinated macaques. Both readouts revealed responses induced in the BCG vaccinated macaques to be of greater magnitude than those made by macaques which did not receive a BCG vaccination before challenge. A rapid increase in the IFNγ response was also revealed in the BCG vaccinated animals 2 weeks after tuberculosis challenge. Taken together these observations suggested that BCG vaccinated animals possessed a higher number of cells which produced more IFNγ, more quickly after challenge with *M. tb*, than unvaccinated animals.

The IFNγ response profile in the unvaccinated macaques defined using the SFU readout showed a steady increase in frequency of cells producing IFNγ over the 18 weeks of study, which was in line with the response trend determined using the spot size x quantity readout. In contrast, the SFU readout revealed a peak in the IFNγ response profile in the BCG vaccinated animals 12 weeks after challenge that was not detected in the profile derived from the spot count x size analysis. This suggested that at week 12, a large number of responding cells were present in the circulation, but individual cells were capable of producing smaller quantities of cytokine than cells assessed at other times after infection. 

## 3. Experimental Section

### 3.1. *In Vivo* Studies

Sixteen UK-bred cynomolgus macaques of Mauritian lineage were selected for Study 1 and twelve cynomolgus macaques of Chinese origin were selected from a UK Home Office approved breeding colony for Study 2. All Animals were housed in the UK according to Home Office (UK) guidelines and were sedated by intramuscular (i.m.) injection with ketamine hydrochloride (10 mg/kg) (Ketaset, Fort Dodge Animal Health Ltd, Southampton, UK) for all procedures requiring removal from their cages. All procedures involving animals were approved by the Ethical Review Committee of the Health Protection Agency, Porton, UK. None of the animals had been used previously for experimental procedures. A PRIMAGAM (Biocor, CSL, USA) test kit was used to demonstrate NHPs were naïve in terms of prior exposure to mycobacterial antigens.

Twelve Mauritian (study 1) and 6 Chinese cynomolgus macaques (study 2) were vaccinated with BCG Danish strain 1331 (Statum Serum Institute, Copenhagen, Denmark). Animals were immunised intradermally into the upper left arm with 100 µL BCG and the viability of the BCG vaccine was confirmed to be within the expected range for the batch (data not shown). 

Twenty-one weeks after vaccination with BCG, the 6 Chinese cynomolgus macaques together with the 6 unvaccinated animals from study 2 were challenged by the aerosol route with 75 CFU *M.tb* (Erdman KO1) using procedures described previously [7,11] then monitored for 28 weeks. Animals were assessed daily for changes in behaviour, and sedated every 2 weeks for changes in clinical parameters including, body weight, temperature, red cell haemoglobin concentration and erythrocyte sedimentation rate, and for collection of blood samples. In this period, disease progressed in 3 of the 6 unvaccinated macaques to levels which met the set humane endpoint criteria and animals were euthanised at weeks 5, 7 and 10 after challenge. All remaining animals successfully controlled disease levels during the post-challenge follow-up period and clinical parameters remained within normal limits. 

Blood samples were collected at 2 weekly intervals from all animals throughout both studies for immune assessment, and PBMCs were isolated using a density gradient centrifugation by standard procedures [7]. 

### 3.2. ELISPOT Assay

The frequency and size of IFNγ producing cells in PBMCs induced by stimulation with mycobacterium-specific antigens was measured using a monkey IFNγ ELISPOT kit from Mabtech (Nacka, Sweden). PVDF 96-well plates (Millipore, Watford, UK) were coated with IFNγ capture antibody. Cells at a concentration of 2 × 10^5^/well were plated out in triplicate and stimulated with either PPD (10 µg/mL, SSI, Copenhagen, Denmark) or Phorbol 12-myristate 13-acetate (PMA) (Sigma-Aldrich Dorset, UK) (100 ng/mL), and Ionomycin (CN Biosciences, Nottingham, UK) (1 µg/mL) as a positive control or left unstimulated. After overnight incubation at 37 °C, cells were washed off and a biotintylated anti-IFNγ secondary antibody added to bind to the released IFNγ. Spots were developed using Streptavidin-HRP enzyme and freshly prepared 5-Bromo-4-Chloro-3-Indolyl Phosphate/Nitro Blue Tetrazolium (BCIP/NBT) substrate (Mabtech, Nacka, Sweden) combination. Spot forming units were counted and average spot areas measured using AID CADAMA ELISPOT reader and software (CADAMA, Stourbridge, UK). 

### 3.3. ELISPOT Analysis

Determinations from duplicate tests were averaged. Data were analysed by subtracting the mean number of spots in the wells with cells and medium-only from the mean counts of spots in wells with cells and antigen. SFU was calculated as the frequency per 10^6^ cells PBMC. Spot size was incorporated into the analysis by multiplying the spot counts by the average spot size per well. Response profiles were plotted using Sigmaplot version 10 (Systat Software Inc, Hounslow, UK). 

### 3.4. Statistical Analysis

To compare the immune response profiles induced in animals by vaccination, the area under the curve (AUC) for each response was calculated using Sigmaplot version 10 (Systat Software Inc, Hounslow, UK) for each animal. The area under the curves was determined for each of the individual animals in each test group. The median AUC was calculated for each group and compared using a Mann Whitney test using Minitab, version 15 (Minitab Ltd, Coventry, UK). Differences of < P = 0.05 were considered to be significant.

## 4. Conclusions

The ELISPOT assay with its traditional SFU readout provides an important tool with proven value in the investigation of the immune response elicited following vaccination or infection. In this study this approach has demonstrated the induction of an increased frequency of cells capable of secreting IFNγ in response to stimulation with mycobacterium-specific antigen in the peripheral blood of macaques, both after vaccination with BCG, and infection with *M.tb*, in line with previous reports [7,12]. The SFU readout clearly demonstrated the response to infection with tuberculosis to be significantly greater than that seen before infection in both BCG-vaccinated and unvaccinated animals. However, at lower frequencies of responding cells, such as those seen after vaccination, the discriminatory power of the SFU readout was reduced to a level such that the response profile defined in Chinese cynomolgus macaques lacked statistical power, or failed to demonstrate a vaccine-induced response in Mauritian cynomolgus macaques.

Consideration of spot size in an ELISPOT assay can provide an additional measure of the immune response. Schlingmann *et al*., [13] have previously reported a transient increase in spot size in an ELISPOT assay after vaccination in a study of the response to vaccinia in human volunteers. Similarly in this study, spots of increased size were observed in assays performed with PBMC collected from Mauritian cynomolgus macaques after vaccination with BCG.

The added value provided by inclusion of the spot size parameter into the ELISPOT readout was found to be more important when responses were lower, *i.e.*, after BCG vaccination, where this approach was shown to improve the ability to discriminate between responses in BCG-vaccinated and unvaccinated subjects. Use of spot size x count revealed clear IFNγ responses to BCG vaccination in both Chinese cynomolgus macaques and Mauritian cynomolgus macaques detectable until at least 18 weeks after vaccination, at the point that the studies ended, which were similar to those reported in rhesus macaques [7]. 

The spot size x spot count readout provides an estimation of both the quantity of IFNγ produced per cell as well as the number of cells producing IFNγ which gives further insight into the nature of the immune response. This readout revealed that not only was the response over the first 6 weeks after TB infection quicker, and the magnitude greater in BCG vaccinated animals, than that seen in previously unvaccinated animals, but also the spots were larger in size. The speed of response, and larger spot sizes early after infection may be indicative of the induction of a secondary response primed by BCG. The spot sizes in assays of the unvaccinated animals were also observed to increase with time after infection to a level that was similar to those seen in BCG vaccinated subjects after infection. This increase in size may also provide evidence of the development of a secondary response as the unvaccinated animals respond to infection. Further work would be required before spot size could be confirmed as an indicator of a secondary response to show that responding cells are from the same population (CD4^+^ T cells) as it is possible that PPD (an undefined antigen preparation) is inducing a lesser amount of IFNγ production from cells other than CD4 T-cells (e.g., gammadelta, NKT or NK cells). Cell size may not be the only factor that influences spot size, as the efficiency with which cytokine can be produced in response to stimulation may also be important. This could be further investigated by measuring the quantity of cytokine released into culture supernatant following antigenic stimulation.

Inclusion of a measure of the spot size into the readout of the ELISPOT assay has shown that useful information can be obtained from spot size measurements, and this could be included in future studies to enhance the information obtained about the IFNγ and Th1 response. An evaluation of the IFNγ response determined using the refined ELISPOT readout as a potential correlate of protection against tuberculosis, or as a biomarker predictive of the progression of tuberculosis disease, should be investigated further.

## References

[1] World Health Organisation (2011). Tuberculosis Facts.

[2] Dye C., Dye C., Scheele S., Dolin P., Pathania V., Raviglione M.C.  (1999). Consensus statement. Global burden of tuberculosis: Estimated incidence, prevalence, and mortality by country. WHO Global Surveillance and Monitoring Project. J. Am. Med. Assoc..

[3] Corbett E.L., Marston B., Churchyard G.J., de Cock K.M. (2006). Tuberculosis in sub-Saharan Africa: Opportunities, challenges, and change in the era of antiretroviral treatment. Lancet.

[4] Colditz G.A., Brewer T.F., Berkey C.S., Wilson M.E., Burdick E., Fineberg H.V.,  Mosteller F. (1994). Efficacy of BCG vaccine in the prevention of tuberculosis. Meta-analysis of the published literature. J. Am. Med. Assoc..

[5] Hussey G., Hawkridge T., Hanekom W. (2007). Childhood tuberculosis: Old and new vaccines. Paediatr. Respir. Rev..

[6] Newport M., Levin M. (1999). Genetic susceptibility to tuberculosis. J. Infect..

[7] Sharpe S.A., Mc Shane H., Dennis M.J., Basaraba R., Gleeson J.F., Hall G.A., McIntyre A., Clark S., Gooch K., Beveridge N.E.R. (2010). Establishment of an aerosol challenge model of tuberculosis in rhesus macaques and an evaluation of endpoints for vaccine testing. Clin. Vaccine Immunol..

[8] Vordermeier H.M., Vordermeier H.M., Chambers M.A., Cockle P.J., Whelan A.O., Simmons J., Hewinson R.G. (2002). Correlation of ESAT-6-specific gamma interferon production with pathology in cattle following Mycobacterium bovis BCG vaccination against experimental bovine tuberculosis. Infect. Immunity.

[9] Chee C.B.E., Gan S.H., KhinMar K.W., Barkham T.M., Koh C.K., Liang S., Wang Y.T. (2008). Comparison of sensitivities of two commercial gamma interferon release assays for pulmonary tuberculosis. J. Clin. Microbiol..

[10] Beveridge N.E.R., Fletcher H.A., Hughes J., Scriba T.J., Pathan A.A., Minassian A., Sander C.A., Whelan K.T., Dockrell H.M., Hill A.V.S. (2008). A Comparison of IFNγ detection methods used in tuberculosis vaccine trials. Tuberculosis.

[11] Sharpe S.A., Eschelbach E., Basaraba R.J., Gleeson F., Hall G.A., McIntyre A., Williams A., Kraft S.L., Clark S., Gooch K. (2009). Determination of lesion volume by MRI and stereology in a macaque model of tuberculosis. Tuberculosis.

[12] Lai X., Shen Y., Zhou D., Sehgal P., Shen L., Simon M., Qiu L., Letvin N.L., Chen Z.W. (2003). Immune biology of macaque lymphocyte populations during mycobacterial infection. Clin. Exp. Immunol..

[13] Schlingmann T.R., Shive C.L., Targoni O.S., Tary-Lehmann M., Lehmann P.V. (2009). Increased per cell IFN-γ productivity indicates recent *in vivo* activation of T cells. Cell Immunol..

